# BGC-XPLORER: Exploring Biosynthetic Gene Clusters with Gene-Level Functional Annotation and AI-Assisted Summarization

**DOI:** 10.3390/cimb48101091

**Published:** 2026-10-21

**Authors:** Vesselin Baev, Elena Apostolova, Mariyana Gozmanova, Ivan Iliev, Velizar Gochev, Galina Yahubyan

**Affiliations:** 1 Department of Molecular Biology, Faculty of Biology, University of Plovdiv, Tzar Assen 24, 4000 Plovdiv, Bulgaria; eapostolova@uni-plovdiv.bg (E.A.); mariank@uni-plovdiv.bg (M.G.); gyahubyan@uni-plovdiv.bg (G.Y.); 2 Department of Biochemistry and Microbiology, Faculty of Biology, University of Plovdiv, Tzar Assen 24, 4000 Plovdiv, Bulgaria; iziliev@uni-plovdiv.bg (I.I.); vgochev@uni-plovdiv.bg (V.G.)

**Keywords:** biosynthetic gene clusters, genome mining, *Streptomyces albidoflavus*, functional annotation, workflow, caller consensus, MIBiG, AI-assisted summarization

## Abstract

Turning one newly sequenced bacterial genome into a view of its biosynthetic potential still means running several cluster detectors and annotation tools and comparing their output by hand. We present BGC-XPLORER, a containerized Snakemake workflow for a single assembled bacterial genome, deployed locally with Docker and operated through a browser. It annotates the genome with Bakta, runs antiSMASH, GECCO and DeepBGC, and groups their predictions gene-by-gene, defining a locus by the genes that at least two callers agree on. Each locus carries gene-resolved evidence from Bakta, eggNOG and dbCAN, ARTS self-resistance hits and MIBiG comparisons in an interactive report with searchable tables and annotated gene maps. Against 42 MIBiG reference loci in four actinomycete genomes from two genera, the grouping recovered 41, at least as many as any single caller in every genome. Applied to the newly sequenced soil isolate *Streptomyces albidoflavus* STR-S001 (GenBank CP158056), the workflow reported 49 loci, of which 27 had support from at least two callers, 22 a MIBiG comparison and five an ARTS known-resistance hit. An optional, remotely generated AI summary of a single locus is offered as an aid to reading the report. The workflow is not a replacement for laboratory verification but a complementary step that precedes it.

## 1. Introduction

Whole-genome sequencing of bacteria has become a routine laboratory step, and rapid, taxon-independent annotation converts an assembled isolate genome or a metagenome-assembled genome (MAG) into standardised gene and protein sets [1]. Genome-mining resources have scaled accordingly: precomputed cluster predictions are available for tens of thousands of bacterial genomes [2], and a survey of approximately 170,000 genomes and 47,000 MAGs has mapped biosynthetic diversity across the bacterial tree [3]. Because the enzymes of a bacterial specialised metabolic pathway are usually encoded by physically clustered genes, such biosynthetic gene clusters (BGCs) can be identified directly from an assembly, so that the metabolic potential of a strain is assessed before any fermentation or chemical work [4]. For biotechnology this is most consequential in the actinomycetes: genus-level comparison indicates that *Streptomyces* encodes by far the largest biosynthetic diversity among bacteria [3], and actinobacteria remain the historically dominant producers of antibiotics and other bioactive compounds [5]. The same analysis estimated that only about 3% of the natural products potentially encoded in bacterial genomes have been identified and characterised [3], and a further fraction of clusters is transcriptionally silent under standard cultivation [6]. Since 4.71 million deaths were associated with bacterial antimicrobial resistance in 2021 and 8.22 million associated deaths are forecast for 2050 [7], while undirected screening of soil actinomycetes repeatedly returns already-described compounds [5], computational selection of promising clusters has become a key step in actinomycete-based discovery.

Bioinformatic detection of BGCs is usually served by several widely adopted callers—the rule-based antiSMASH, whose version 8 covers 101 cluster types [4], the deep-learning DeepBGC [8], the conditional-random-field GECCO and InterPro-based SanntiS, both still preprints [9,10]—but for a discovery project the operative unit is the pipeline built around them. Post-processing toolboxes such as Palantir refine and reformat antiSMASH output for downstream use [11]. Downstream tools add further per-cluster evidence computed independently of detection: OOPS weights clusters by sequence-derived and taxonomic criteria [12], ARTS (Antibiotic Resistant Target Seeker) flags self-resistance evidence from essential housekeeping genes [13,14,15], and the recent novelBGC framework combines prioritisation (preprint) [16]. Comparative workflows group clusters into families or retrieve homologues—BiG-SCAPE with CORASON [17], BiG-SLiCE [18], the derived BiG-FAM database [19], and cblaster [20] with its web front end CAGECAT [21]. Systematic multi-stage workflows orchestrate these steps: BGCFlow chains data selection, genome mining and comparative analysis over bacterial pangenomes [22], and nf-core/funcscan provides an analogous Nextflow screening workflow [23]. Domain-specialised pipelines extend the same design to other questions, among them gutSMASH for primary metabolic gene clusters of the gut microbiota [24], BiG-MAP for cluster abundance and expression in metagenomic and metatranscriptomic data [25], and SeMPI 2.0 for polyketide and non-ribosomal peptide scaffold prediction with natural-product database screening [26]. Pipeline outputs populate repositories such as the antiSMASH database [2], IMG-ABC [27] and BGC Atlas [28].

Where more than one detector is run, predictions are reported side-by-side; a recent benchmarking effort documents that the outputs of genome-mining tools differ substantially and are difficult to compare (preprint) [29], so the comparison usually is made manually by the user. antiSMASH annotates the genes inside its own regions with protein domains and smCOG-based functional categories [4], but orthology-based functional annotation, carbohydrate-active enzyme families and self-resistance evidence are computed by separate tools at the genome or pangenome level, and loci called only by other detectors receive no gene-level annotation at all; the transporters, regulators, tailoring enzymes and carbohydrate-active enzymes inside a given cluster are therefore not brought together, with their functional annotation, for the analyst assessing it. The available evidence layers also remain siloed from one another: OOPS, which scores sequence-derived and taxonomic statistics [12], ARTS, which scores self-resistance [13,14,15], and MIBiG (Minimum Information about a Biosynthetic Gene cluster) [30], each capturing one facet of the evidence in its own output rather than a shared, per-locus representation. Finally, the systematic workflows are built for command-line use and for genome collections rather than for one newly sequenced isolate; the repositories cover only deposited genomes, and the tool versions, database releases and parameters that determine a result are seldom recorded with it.

BGC-XPLORER, a containerized application that treats a single bacterial genome as the unit of analysis, accepts an assembled bacterial genome in FASTA format through a locally deployed, browser-based interface, annotates it with Bakta [1], runs antiSMASH [4], GECCO [9] and DeepBGC [8], and groups their predictions gene-by-gene. Onto those outputs the workflow projects gene-resolved evidence—Bakta gene products [1], eggNOG annotations and functional categories [31], and, from dbCAN, carbohydrate-active enzymes (CAZymes) with predicted substrates [32]—together with ARTS self-resistance hits [14] and MIBiG comparisons [30]. Results are returned as an interactive report with detailed gene maps and tables. The workflow is implemented in Snakemake [33] and distributed as one container image. An optional AI summary restates the evidence computed for an individual cluster, with every sentence checked against that evidence. The workflow thus provides merged detector outputs rather than parallel tool reports, and integrates cluster-level data with gene-level annotation and functional features in interactive gene maps. We validate the grouping against MIBiG loci in four reference genomes from two genera and apply the workflow to a newly sequenced soil isolate.

## 2. Materials and Methods

### 2.1. Architecture, Deployment and Input

BGC-XPLORER is a Snakemake v9.23.1 workflow [33] distributed as one OCI image (micromamba v2.0.5) in which every tool occupies an isolated conda environment. A NiceGUI v3.14.0 browser interface [34] accepts an assembled bacterial genome as nucleotide FASTA and registers it as a Snakemake target, so completed outputs are reused on restart. Reference databases are mounted from the host and checked against a versioned manifest at start-up. The version described here is release v1.2.2 (https://github.com/vebaev/BGC-XPLORER/releases/tag/v1.2.2 accessed on 14 October 2026).

For every run the workflow writes a provenance record, provenance.json, with the results. It lists the workflow version and commit, the version of each tool, the configuration and the SHA-256 checksums of the input files and of every reference database file, with API keys, passwords and tokens removed, so that a reader can check that a later run used the same software, databases and input (File S1 for the run reported here).

### 2.2. Genome Annotation

Annotation uses Bakta v1.12.0 [1] with the light or full database. Bakta provides the gene set onto which all evidence is projected and on which caller support is counted. antiSMASH runs on the Bakta GenBank file and therefore on Bakta’s genes, whereas GECCO and DeepBGC run on the nucleotide FASTA and call genes themselves; their gene calls are used only inside each caller to produce its predicted regions. Each predicted region enters the grouping as a genomic interval, and every Bakta coding sequence (CDS) that the interval overlaps receives that caller’s support, so no matching between the callers’ gene calls and Bakta genes is needed, and a caller’s gene that spans two Bakta genes, or none, does not affect the grouping. eggNOG-mapper, dbCAN and ARTS work on the Bakta proteins and annotation.

### 2.3. Cluster Detection

Three detectors are run, but not all of them on the Bakta gene set. antiSMASH v8.0.4 [4] analyses the Bakta GenBank file against its own reference data, including Pfam 35.0 [35], with KnownClusterBlast and ClusterCompare against MIBiG enabled; GECCO v0.10.3 [9] applies conditional random fields to the same genome; and DeepBGC v0.1.31 [8] runs on the nucleotide FASTA with Prodigal v2.6.3 [36] and Pfam-A 31.0 models [35]. HMMER3 [37] is used throughout. GECCO and DeepBGC therefore contribute genomic intervals rather than gene calls; those intervals are resolved to Bakta coding sequences by overlap in the grouping step (Section 2.5), and no matching between the callers’ own gene models and Bakta genes is performed.

### 2.4. Gene-Level, Resistance and Dereplication Evidence

ARTS [14] performs target-directed genome mining on the Bakta GenBank file with the Actinobacteria reference set as default and the antiSMASH output as cluster context. eggNOG-mapper v2.1.13 [31] annotates the Bakta proteins in DIAMOND mode [38], and dbCAN v5.2.9 [32] runs in easy_substrate protein mode on the Bakta proteins and GFF3, adding CAZymes, CAZyme gene clusters and predicted substrates. A CAZyme family and the CAZyme gene category are taken only from dbCAN’s recommended result, which requires agreement of at least two of its three methods (HMMER, dbCAN-sub and DIAMOND); a family found by a single method is shown in the gene tooltip and marked as not recommended. A gene is flagged as an annotation conflict when dbCAN’s recommended result assigns it a CAZyme family while a caller marks it as a biosynthetic core gene. MIBiG comparisons [30] are read from the structured antiSMASH output by KnownClusterBlast, ClusterCompare against MIBiG and CompariPPson for RiPPs (ribosomally synthesised and post-translationally modified peptides), and one representative comparison is kept per locus by a fixed priority: CompariPPson at ≥80% peptide similarity, then KnownClusterBlast (accepted only if at least one core gene is shared), then CompariPPson below 80%, then ClusterCompare; ties are broken by the number of shared core genes, then of matched genes, then by the score. A gene is recorded as a biosynthetic core gene when a Pfam family with a direct role in scaffold formation is assigned to it. Ten families are used: PF00109 and PF02801 (beta-ketoacyl synthase and its C-terminal domain), PF00698 (acyltransferase), PF00501 (AMP-binding enzyme), PF00550 (phosphopantetheine carrier protein), PF00975 (thioesterase), PF01397 and PF03936 (terpene synthase and its metal-binding domain), and PF05147 and PF14028 (lanthionine synthetase C-terminal domain and lanthipeptide dehydratase). Where no such domain is assigned, a fallback accepts a Bakta product name containing one of eleven phrases that name a scaffold-forming enzyme, among them non-ribosomal peptide synthetase, polyketide synthase, terpene synthase and lanthipeptide synthetase; generic terms such as synthetase, oxidase, transferase and cyclase are excluded, because they also match tailoring and housekeeping enzymes. The ten families are a deliberately conservative subset: aminoglycoside, nucleoside and several RiPP machineries will not raise the flag, so a locus without a core gene is not evidence that no scaffold enzyme is present.

### 2.5. Grouping of the Outputs

Predictions on the same contig are grouped by caller agreement at the gene level rather than by spanning regions. Every Bakta CDS is a voting unit, and a caller votes for a gene when any of its predicted regions overlaps the gene by at least 1 bp. A multi-caller locus is a maximal run of consecutive genes each covered by at least two of the three detectors. There is no gap tolerance: a gene covered by fewer than two callers ends the run, and a new locus starts where agreement resumes. There is no minimum length: a single gene covered by two callers forms a locus. A prediction whose genes fall into two agreed runs becomes a member of both loci, so a large region such as an antiSMASH hybrid region is split where the other callers’ support breaks, and neighbouring predictions whose genes lie in one run are joined. A prediction that no agreed run covers is kept whole, so evidence from a single caller is also recorded and no prediction is discarded. The thresholds are configurable (min_callers_per_gene, default 2; min_genes_per_locus, default 1; min_gene_overlap_fraction, the share of a gene a prediction must cover to vote, default 0), and the defaults reproduce the rule described here. Raising min_gene_overlap_fraction above zero withdraws the vote of a prediction that merely clips the first or last gene of a run, which contracts the agreed span by that gene; Section 3.4 gives a worked example. The value used is recorded in the provenance record of every run, so a reported span can always be traced to the setting that produced it. Counting support per gene rather than comparing regions removes the need to judge whether two callers drew the same boundaries, which they rarely do: antiSMASH extends beyond the core genes, GECCO cuts at coding-sequence edges and DeepBGC at domain boundaries. The coordinates reported for a locus are therefore the span of the agreed genes, while the gene tables and gene maps cover the union of the contributing predictions, so that no gene reported by any caller is lost; where the two differ, the gene map shades the agreed span inside the wider window. The number of callers supporting each individual gene is written to a separate table. Each group is additionally checked for biosynthetic core genes, which are recorded as supporting evidence but never merge regions. Each locus carries its supporting callers, ARTS known-resistance and DUF (domain of unknown function) hit counts, representative MIBiG comparison, and predicted classes and activities. GECCO and DeepBGC are both machine-learning callers built on Pfam domain annotations, so their agreement is partly correlated and is weaker evidence than agreement involving antiSMASH; loci supported only by GECCO and DeepBGC are therefore reported as a separate category.

### 2.6. Report

Summary tables are written as TSV and rendered into a self-contained HTML report in which all loci appear in one searchable table with gene maps. Class labels from the three callers are mapped onto the MIBiG compound classes (NRP, Polyketide, RiPP, Terpene, Saccharide, Alkaloid, Other and Unknown), so that NRPS, NRP and NRPS-like count as NRP and T1PKS and Polyketide as Polyketide; the caller’s original label is kept for display. A locus carries the classes of all its contributing predictions and is counted once under each of them, so the class counts can exceed the number of loci. Activity labels are DeepBGC predictions made from Pfam domain content, not assay results, and are counted separately.

For a locus of interest the user may request an optional AI summary from a remotely served language model; the default model is NVIDIA/nemotron-3-ultra-550b-a55b served by NVIDIA NIM, and the user supplies their own API key and may choose another model. The model is not asked to interpret the raw evidence. The workflow first computes numbered facts for the locus from its own outputs: the callers, the gene roles, the NRPS/PKS modules and substrate predictions and the KnownClusterBlast gene pairs from antiSMASH, and the MIBiG 4.0 entry of the closest known cluster [30]. The model words these facts in three short parts (what the cluster is, what it contains and what it resembles) and cites the facts of every sentence. Each sentence is then checked by the software: its numbers, locus tags, accessions, callers, substrates and compound names must come from the facts it cites, a count must count the same category, a gene’s attributes must come from a fact about that gene, and interpretive wording is rejected. A sentence that fails is removed, and the report states how many were removed; definitions of technical terms come from a fixed glossary added by the software. Any interpretation is confined to a separate box labelled “Hypothesis (AI-generated, not verified)”. The check establishes that a summary restates the evidence faithfully, not that the evidence itself is correct. The prompt and the generation parameters are fixed (temperature 0.2, top-*p* 0.95, at most 2500 tokens, seed 42, reasoning off, structured JSON output), and every stored summary records the model, prompt version, parameters, date and an SHA-256 fingerprint of the full request. The report states, beside the button and with the model’s name, under every summary, that AI models can make mistakes; no result reported here depends on the AI layer.

### 2.7. Validation Against MIBiG

The grouping was validated on four reference genomes from two genera: *Streptomyces coelicolor* A3(2) (NC_003888.3, with plasmids SCP1 and SCP2), *S. albidoflavus* J1074 (CP004370.1), *S. avermitilis* MA-4680 (NC_003155.5, with plasmid SAP1) and *Salinispora tropica* CNB-440 (CP000667.1). Every active MIBiG 4.0 entry [30] was aligned to each genome with megablast [39] and accepted at ≥95% identity over ≥90% of its length; locus coordinates were taken from the alignment rather than from the deposited record, and entries at the same place were collapsed, giving 42 reference loci (18, 7, 11 and 6 per genome). antiSMASH, GECCO and DeepBGC alone and BGC-XPLORER were scored from the same workflow run of each genome, with the light Bakta database. Recovery was classified with the BGC-QUAST categories [29] (full: one prediction covers ≥95% of the reference locus; partial: predictions cover 10–95%; missed: <10%), and BGC-QUAST 1.1.0, run in compare-to-reference mode with the MIBiG loci as the reference, served as an independent check. Boundary accuracy was measured as the Jaccard index in bp between the reference locus and the best-matching prediction, together with gene-level precision, recall and F1 inside the recovered locus. Locus-level precision is not reported, because MIBiG does not annotate every biosynthetic gene cluster in these genomes and a predicted locus without a MIBiG counterpart may be a genuine, uncharacterised cluster rather than a false positive. Paired comparisons used the Wilcoxon signed-rank test and a genome-stratified bootstrap (10,000 resamples) of the mean difference. For comparison with side-by-side reporting, the three callers’ predictions were also concatenated into one table without grouping, as the comBGC step of nf-core/funcscan reports them [23]. For each reference locus the Jaccard index of that concatenated table is the arithmetic mean of the Jaccard indices of all its rows overlapping the locus, which is the expected outcome for a user who takes one of the overlapping rows without knowing which is correct; the computation is deterministic and involves no sampling. All prediction sets were scored on the agreed gene span, and BGC-QUAST was run on the same intervals; the wider gene-map window was scored separately, as an arm of its own. A reference locus with no matching prediction was entered with a Jaccard index of zero rather than excluded. Grouping variants were evaluated on the same 42 loci and are reported in Table S5: voting with one, two or three callers per gene, each scored on the agreed span, on the union window and restricted to the multi-caller tier, together with the containment rule of the previous release. The default of two callers per gene was fixed when that rule was released, before the reference set and the scoring script existed, and was not selected on this benchmark. Two of the three callers, GECCO and DeepBGC, were trained with MIBiG clusters as positive examples, so their recovery on a MIBiG-derived reference set is an upper bound rather than an unbiased estimate, and the same caution applies to any arm that includes them. Scripts, reference loci, metrics, tables and the per-locus results are provided in the repository (validation/).

### 2.8. Availability and Installation

BGC-XPLORER is distributed from GitHub at https://github.com/vebaev/BGC-XPLORER (accessed on 14 October 2026) under the MIT Licence and as a versioned container image on the GitHub Container Registry (ghcr.io/vebaev/bgc-xplorer:1.2.2). A new clone retrieves the default branch, release/public-github, and the release described here is checked out with git checkout v1.2.2. Installation consists of copying .env.example to .env, which pins the image version, and running docker compose up -d. The .env file sets the host port (8778 by default), the thread budget, the Bakta database type, the ARTS reference set, automatic database preparation, and the key and model for the optional AI service. Reference databases for Bakta, antiSMASH, DeepBGC, ARTS, eggNOG and MIBiG are installed into the mounted db directory on first start. The reference databases occupy 83 GB with the light Bakta database and 163 GB with the full one, of which eggNOG alone accounts for 59 GB (Table S1). A 320 kb fragment of CP158056.1 with its expected loci is provided in the repository (example/) for testing an installation.

## 3. Results

### 3.1. Web Interface

The interface is a single page served by the locally deployed container (Figure 1). The user supplies a sample name and one uncompressed nucleotide FASTA file (.fa, .fasta or .fna, up to 30 MB) and starts the run; no command-line interaction is required after the one-off installation, and annotation, cluster detection and the report are computed on the host. Only the optional AI summary sends data off the host: the facts computed for the selected locus are sent to the remote model provider, whose terms may allow submitted data to be retained or used for training, which users of unpublished or proprietary genomes should weigh before enabling it. While the workflow runs, the page shows a step counter, progress bar and live log, and on completion links to the standalone HTML report, which can be shared independently.

### 3.2. Biosynthetic Repertoire of a Newly Sequenced Soil Actinomycete Streptomyces albidoflavus STR-S001

BGC-XPLORER was applied to *Streptomyces albidoflavus* STR-S001, an actinomycete isolated from soil. Genomic DNA was sequenced on an Oxford Nanopore Technologies R10.4.1 flow cell with the Rapid Barcoding Kit SQK-RBK114 (Oxford Nanopore Technologies, Oxford, UK); reads were base-called and quality-filtered with Guppy v6.5.7, adapters were trimmed with Porechop v0.2.4 and reads shorter than 1000 bp were discarded. The reads were assembled de novo with Flye v2.9.2 [40] into a single chromosome at a mean depth of 40×, and the assembly was polished with Racon v1.4.21 [41] followed by Medaka v1.8.1. The chromosome is 7,050,834 bp long with a GC content of 73.4%; CheckM v1.1.6 [42] estimates 99.53% completeness and 0.69% contamination. The species assignment rests on average nucleotide identity: FastANI v1.34 [43] gives 99.06% to the type strain *S. albidoflavus* DSM 40455 and 99.09% to NRRL B-1271, the same type strain in another collection, above the 95–96% species threshold. The polished chromosome is deposited in GenBank under accession CP158056.1 and is the sequence analysed here; it was submitted through the web interface under the sample identifier Soil_1, which prefixes every locus identifier. The run with release v1.2.2 and the full Bakta database took 2 h 42 min from the FASTA file to the finished report with 16 threads and a peak resident memory of 11.5 GiB, on a server with two Intel Xeon Gold 6148 CPUs (80 hardware threads) and 376 GB RAM; most of the time is spent in the eggNOG search. With the light Bakta database, in an earlier run on the same server, the workflow took 2 h 03 min and 6.8 GiB (full-database runs of successive releases on this shared server took 2 h 14 min to 2 h 42 min) and reported the same 49 loci with identical coordinates and caller support, at the cost of less-specific gene annotation (248 of the 1764 genes in the loci remained hypothetical proteins, against 58 with the full database). At least eight CPU threads are recommended; the thread budget is set when the container is started, together with the API key, model and port.

Once the report is complete it opens with the sample header and an At a Glance panel (Figure 2). For strain STR-S001 the workflow grouped the predictions of the three detectors into 49 loci on the single chromosome, of which 27 are supported by at least two callers and 22 by a single caller; 22 carry a representative MIBiG comparison and five overlap an ARTS known-resistance record. Two charts summarise the loci. Over the 49 loci there are 56 class assignments (Unknown 16, NRP 10, RiPP 10, Polyketide eight, Terpene eight, Other three and Saccharide one), and 13 loci carry more than one class; Unknown means that no caller assigned a class. DeepBGC predicts an activity for 18 loci (antibacterial 17, cytotoxic 2; Soil_1_32 carries both labels); these are predictions from Pfam domain content, not assay results. The complete report is provided as File S2.

The loci fall into three kinds. Nineteen agree with antiSMASH, that is, antiSMASH and at least one other caller support them (all three callers 13, antiSMASH and GECCO five, antiSMASH and DeepBGC one), and all 19 contain a biosynthetic core gene; the known candicidin, surugamide, desferrioxamine, ectoine and geosmin clusters are among them. Eight are supported by GECCO and DeepBGC without antiSMASH, and three of them with a core gene; because both callers are built on Pfam domain annotations, their agreement is weaker evidence than agreement involving antiSMASH, and these loci are reported as a category of their own. The remaining 22 loci rest on a single caller (DeepBGC 13, GECCO six, antiSMASH three). Seven of them contain a biosynthetic core gene—three antiSMASH-only loci (two RiPP regions and a terpene region whose closest MIBiG comparison is the gaudimycin/rabelomycin cluster) and four DeepBGC-only loci—and are the ones we suggest examining first; the other 15 carry no core gene and may be fragments or false positives. Table S2 lists all 49 loci with their coordinates, callers, genes covered by at least two callers, core genes, MIBiG comparison, ARTS hits, classes and activity predictions.

The MIBiG comparison names the closest characterised product for each of the 22 matched loci. Several loci are recovered with KnownClusterBlast on a substantial number of genes: candicidin (BGC0000034; 23 genes matched, eight of them core), surugamide A/D (BGC0001792; 23 and six) and cyclofaulknamycin (BGC0002358; 24 and two), together with hopene (BGC0000663; ten and four) and the smaller desferrioxamine B (BGC0000941), ectoine (BGC0000853), geosmin (BGC0001181), albaflavenone (BGC0000660) and naringenin (BGC0001310) clusters. An RiPP locus is matched instead by CompariPPson, at 81.8% peptide similarity to the lanthipeptide SRO15-2212 (BGC0000553). Other loci match at lower scores or only through ClusterCompare, among them bovienimide A, a non-ribosomal peptide from *Xenorhabdus bovienii* (BGC0002135); cyclothiazomycin B1, a ribosomally synthesised peptide from *Streptomyces mobaraensis* (BGC0001145); and colicin V, a ribosomally synthesised peptide from *Escherichia coli* (BGC0001555) [30]. A match of this kind identifies chemistry that is already characterised and is the point at which dereplication can be applied; where the closest relative comes from a distantly related organism, the shared feature is more often the biosynthetic or export machinery than the final compound.

dbCAN detects 75 CAZyme gene clusters (CGCs) in the genome and predicts a substrate for 17 of them, which are listed in the CGC tab of the report; the substrates are most often xylan (five), beta-glucan (four) and starch or alpha-glucan (four), with chitin, cellulose, levoglucosan and host glycan also represented, a profile pointing at plant litter and fungal cell wall as the carbon sources the strain is equipped to use and consistent with a soil saprophyte profile. Carbohydrate gene clusters are a different class of locus from biosynthetic gene clusters and are not presented as such; they are carried as an additional layer of annotation describing the analysed strain.

### 3.3. The Evidence Table

All clusters are presented in a single searchable table with two tabs, BGC (49) and CGC (CAZyme gene clusters, 17), preceded by a legend naming each column (Figure 3); a third tab offers the underlying tables for download. A row gives the locus identifier, the coordinates of the agreed span with its contig and length, the number of annotated genes, the callers that predicted it, the counts of ARTS known-resistance and DUF hits, the closest characterised MIBiG cluster (expandable to the method and product behind the match), and the predicted classes with any activity label as a badge; a button opens the annotated gene diagram. Rows are ordered by genomic coordinate. There are no confidence tiers or rankings; selection, sorting and filtering are left to the reader.

### 3.4. Gene-Resolved Inspection of a Locus

Selecting a cluster opens its detailed gene map (Figure 4). The example shown, Soil_1_13 (Soil_1_14 in the original submission), also shows how a locus is delimited. antiSMASH reports contig_1:1,326,997–1,390,107, GECCO 1,336,178–1,389,372 and DeepBGC 1,333,732–1,379,192. The agreed run starts at CJLEIP_01135, the first gene covered by two callers (antiSMASH and DeepBGC; CJLEIP_01134 is covered by antiSMASH alone), and ends at CJLEIP_01164, the last gene covered by antiSMASH and GECCO. The locus is therefore reported at contig_1:1,332,869–1,390,082 (57,214 bp, 30 genes, 20 of them covered by all three callers), while the map is drawn across the union of the three predictions, contig_1:1,326,997–1,390,107 (36 genes), and shades the agreed span inside it; DeepBGC and GECCO touch the two end genes by only 4 bp each, 0.46% of CJLEIP_01135 (867 bp) and 0.56% of CJLEIP_01164 (714 bp); setting min_gene_overlap_fraction above 0.0056 withdraws both votes, so each gene falls to a single caller and the agreed span contracts to contig_1:1,333,732–1,389,372 (55,641 bp), one gene shorter at each end, while both genes remain drawn in the map with a caller count of one. The locus is a non-ribosomal peptide candidate with a DeepBGC antibacterial prediction, and its closest characterised relative is cyclofaulknamycin (BGC0002358), matched on 24 genes, two of them core. Its genes are drawn to scale and coloured by category—three biosynthetic core genes, four tailoring enzymes, four genes with resistance evidence, eleven transporters, six regulators, one carbohydrate-active enzyme, one mobile element and six others—and the accompanying table gives, for every locus tag, the Bakta annotation, the eggNOG functional description and the dbCAN family. The assembly line is readable directly from those rows: two large *dltA* genes (CJLEIP_01147 and CJLEIP_01149, 10.2 and 12.5 kb), both placed by eggNOG among non-ribosomal peptide synthetase modules, flank an MbtH family protein (CJLEIP_01148). A class A beta-lactamase-related serine hydrolase (CJLEIP_01146) precedes them, and an L-asparagine oxygenase carrying clavaminate synthase activity (CJLEIP_01150), a cytochrome P450 (CJLEIP_01152) and a thioesterase (CJLEIP_01154), the third core gene, follow. The same rows show an annotation conflict: Bakta names CJLEIP_01147 and CJLEIP_01149 D-alanine--poly(phosphoribitol) ligases and eggNOG places them among non-ribosomal peptide synthetase modules, while dbCAN’s DIAMOND search alone assigns them to glycosyltransferase family GT1. dbCAN does not recommend this single-method assignment, so the workflow shows it in the tooltip but uses neither the family nor the CAZyme category; the value of the table is that such a conflict is visible on one row, not that every source is right.

### 3.5. Optional AI Summary

For the selected locus the workflow assembles the numbered facts described in Section 2.6, and the model’s three-part summary is shown with every sentence checked against them; hovering over a sentence shows the facts behind it, and the hypothesis, if any, appears in a separate, labelled box. For Soil_1_13, the summary returned by NVIDIA/nemotron-3-ultra-550b-a55b states, for instance, that the two NRPS genes CJLEIP_01147 and CJLEIP_01149 hold six modules, for four of which antiSMASH predicts phenylalanine, valine and two threonines, that three modules carry an epimerization domain, and that the closest known cluster is cyclofaulknamycin (BGC0002358); the hypothesis box adds that the cluster may produce a cyclofaulknamycin-like non-ribosomal peptide modified by oxidation and glycosylation. To assess the layer we generated summaries for ten loci of STR-S001, chosen to cover close, moderate, distant and absent MIBiG matches and loci found by one, two or three callers, and read every sentence against the evidence table (File S3). Of the 89 sentences the model wrote, the check removed 12: ten correct but citing a word or number that was not in their facts, one built from genes listed only for the hypothesis and one worded interpretively. Of the 77 shown, 74 were correct, three were imprecisely worded and none were wrong. The model recommended in the previous release, NVIDIA/nemotron-3-super-120b-a12b, was retired by its provider during this revision; the evaluation was therefore made with its replacement. The summaries remain an aid to reading the evidence table, not a replacement for it.

### 3.6. Validation of the Grouping Against MIBiG

Against the 42 MIBiG loci in the four reference genomes, BGC-XPLORER detected 41 (recall 0.98), more than any single caller (antiSMASH 40, GECCO 38, DeepBGC 32), and at least as many as any single caller in every genome (Table 1; Table S3). Because a prediction that no agreed run covers is retained as a single-caller locus, this figure is a union property of the three callers; restricted to the loci carrying at least two callers it is 38 of the 42, at a median Jaccard index of 0.482. The one locus recovered here and not by antiSMASH is the SCO-2138 lanthipeptide cluster of *S. coelicolor* A3(2) (BGC0000595), supported by GECCO alone and recovered in full. The one locus it missed, the 5 kb ochronotic pigment cluster of *S. avermitilis*, was missed by all three callers.

Across the 42 paired loci BGC-XPLORER scored the higher Jaccard index for 22, and antiSMASH for 17, with three ties (median 0.524 against 0.458; mean paired difference +0.079, Wilcoxon *p* = 0.12), so the advantage does not reach significance at this sample size; the median favours the grouping in three of the four genomes (Table S3). A reference locus that a prediction set misses is entered with a Jaccard index of zero. GECCO alone drew tighter boundaries (median Jaccard 0.582) but missed four loci, and failed on different loci in each genome.

The comparisons that isolate the grouping rule are made on the same predictions from the same runs. Against the containment rule of the previous release the mean paired difference in Jaccard is +0.168 (*p* = 9.0 × 10^−7^), against voting that accepts a single caller per gene +0.251 (*p* = 1.8 × 10^−12^), and against scoring the same loci on the wider gene-map window rather than on the agreed span +0.229 (*p* = 2.4 × 10^−10^); confidence intervals and the remaining variants are given in Table S5. Fusion runs in the same direction: the grouping fuses two of the 42 reference loci, against four for antiSMASH and five for the containment rule. Both are in J1074, where one prediction spans the adjacent candicidin and antimycin clusters, so recovery category and boundary index must be read together. Against the concatenated table the grouping matched the reference loci more closely than an overlapping row taken without knowing which is correct (+0.054, *p* = 3 × 10^−4^) and reported 226 rows against 400 (Table S4). The recovery status and Jaccard index of every locus are given in the repository (validation/).

## 4. Discussion

Three properties distinguish BGC-XPLORER from the workflows alongside which it is meant to be used. The first is that it reconciles its detectors. Where several callers are run together their predictions are normally reported side-by-side: the comBGC step of nf-core/funcscan concatenates the antiSMASH, GECCO and DeepBGC predictions into one table, one row per prediction per tool, without overlap detection, merging or deduplication [23]; BGCFlow keeps antiSMASH and GECCO as separate outputs and groups whole antiSMASH regions into gene cluster families with BiG-SCAPE and BiG-SLiCE, so its locus borders are those of antiSMASH [17,18,22]; and prioritisation frameworks such as novelBGC score whole antiSMASH regions [16]. Reconciling the callers is left to the user, and whatever is reconciled inherits the borders each detector assigned. BGC-XPLORER resolves every prediction to annotated genes instead and defines a locus by caller agreement gene-by-gene, so the reported interval follows the evidence rather than any one caller’s flanking convention, and the number of callers supporting each individual gene is reported alongside the locus. On the four reference genomes, side-by-side reporting of the same predictions left a median of three overlapping rows per MIBiG locus; taking any one of them, as a user who does not know which is correct would, matched the locus less closely than the BGC-XPLORER locus, and BGC-XPLORER reported 226 rows instead of side-by-side 400; the comparison is defined in Section 2.7 and reported in Section 3.6 and Table S4.

The second is where the functional annotation is placed. antiSMASH annotates every gene inside its own regions with protein domains and smCOG-based functional categories [4], and functional annotation is part of several genome-mining platforms, where it is computed at the genome or pangenome level and reported separately from the clusters themselves [22]. Here the eggNOG orthology assignments (EC number, KEGG orthologue and COG category), the dbCAN carbohydrate-active enzyme families with predicted substrates and the ARTS self-resistance hits, which antiSMASH does not provide, are intersected gene-by-gene with every locus and displayed inside it, including the loci that antiSMASH does not call at all, seven of which carry a biosynthetic core gene (27 such loci in STR-S001; the other 20 carry none and are offered as candidates for inspection rather than as results), so that a locus is read as a list of annotated genes rather than as a class label. Disagreements between sources are made visible rather than resolved silently: a gene that dbCAN’s recommended result assigns to a CAZyme family while a caller marks it as a biosynthetic core gene is flagged as an annotation conflict, with a dashed outline on the gene map, an explanation in the tooltip, a badge in the gene table and a column in the gene table file. Taking CAZyme families only from dbCAN’s recommendation removed every such conflict in STR-S001, where 30 genes had been flagged when a single dbCAN method was accepted, and reduced the genes classed as CAZymes from 86 to 56. The same mechanism carries the carbohydrate-active layer, which sets the biosynthetic repertoire against the substrates the strain is equipped to use.

The third is delivery. A completed run is a single self-contained HTML file carrying the searchable table, the filters, every gene map and the underlying tables for download, so an analysis can be archived as one file or sent to a collaborator, and the provenance record lists the tool versions and database checksums behind it. After the one-off installation on the host machine, no command line is involved for the end user: a FASTA file is uploaded in a browser and the run starts with one click. The optional AI summary only restates the evidence computed for one locus, with every sentence checked against it; it is an aid to reading the evidence table, not a replacement for it.

Workflows of this kind matter most at the point where a newly sequenced genome first has to be made sense of. Annotation and cluster detection are each routine on their own, but turning them into a view of the biosynthetic repertoire of one isolate still means assembling several outputs by hand, and it is that step the workflow removes. BGC-XPLORER does not supersede the detectors it runs, nor the pangenome-scale pipelines built for genome collections; it sits alongside them as an additional workflow for a laboratory working on individual clusters, bringing the output of several callers, gene-level functional annotation and an optional AI summary together for one genome at a time. Its scope is one assembled bacterial genome per run, and the grouping has been validated on four genomes from two genera of actinomycetes; its performance on other taxa remains to be measured. What it returns is predictive throughout. Nothing in the report establishes that a compound is made, and every locus remains a hypothesis to be tested by expression, isolation and chemical characterisation in the laboratory.

Several limitations should be pointed out. Installation requires Docker and 83–163 GB of reference databases, and a machine with at least eight threads is recommended; these are one-off costs, after which the workflow is driven entirely from the browser. We do not provide a native installation, because the container is what fixes the versions of the ten pinned tools and makes the provenance record that lists them meaningful. The intended deployment is a single workstation or a laboratory server on a trusted network. Analysis is restricted to one genome per run, which suits a laboratory working on individual isolates but not a pangenome-scale survey, for which the platforms cited above remain the appropriate choice [22,23]. The present version is developed and tested for whole bacterial genomes; fragmented assemblies, metagenome-assembled genomes and plasmids raise questions it does not yet resolve, such as how contig edges affect the agreed span and how a locus interrupted by a contig break should be reported, and their support is planned for a future release. GECCO, one of the three detectors, is described only in a preprint, and for an earlier version (0.6) than the one run here (0.10.3, with a retrained model and a lower default threshold); its accuracy in this workflow is measured directly by the MIBiG validation, and its version is pinned in the container and recorded in the provenance record. Finally, the optional AI summary depends on a remotely served model. AI models can make mistakes, and the check establishes that a summary restates the facts computed by the workflow, not that the tools’ predictions behind those facts are correct. Reproducibility across models is not claimed, because the user chooses the model and a remote model can change behind the same identifier; the stored record names the model, prompt version and date of every summary. When the AI layer is enabled, the facts computed for the selected locus leave the host for the provider, whose terms may allow submitted data to be retained or used for training, which matters for unpublished or proprietary genomes. This layer is offered only as an optional aid, and none of the results reported here rely on it.

## 5. Conclusions

BGC-XPLORER turns one assembled bacterial genome into a single, inspectable account of its biosynthetic gene clusters: the predictions of antiSMASH, GECCO and DeepBGC are grouped gene-by-gene, each locus carries gene-resolved functional, resistance and dereplication evidence, and the analysis is recorded with the tool versions and databases behind it. On four reference genomes the grouping detected more MIBiG loci than any single caller, a union property that follows from retaining single-caller predictions, and its median boundary agreement with those loci exceeded that of antiSMASH alone without reaching significance across the 42 paired loci (Wilcoxon *p* = 0.12); the comparisons that isolate the agreement rule itself, against the previous grouping rule and against the wider gene-map window, were larger and significant. The workflow is not a substitute for laboratory verification but a complementary step that precedes it, giving the user a structured way to predict and explore the biosynthetic potential of a newly sequenced strain and to decide which loci are worth taking to the bench.

## Figures and Tables

**Figure 1:**
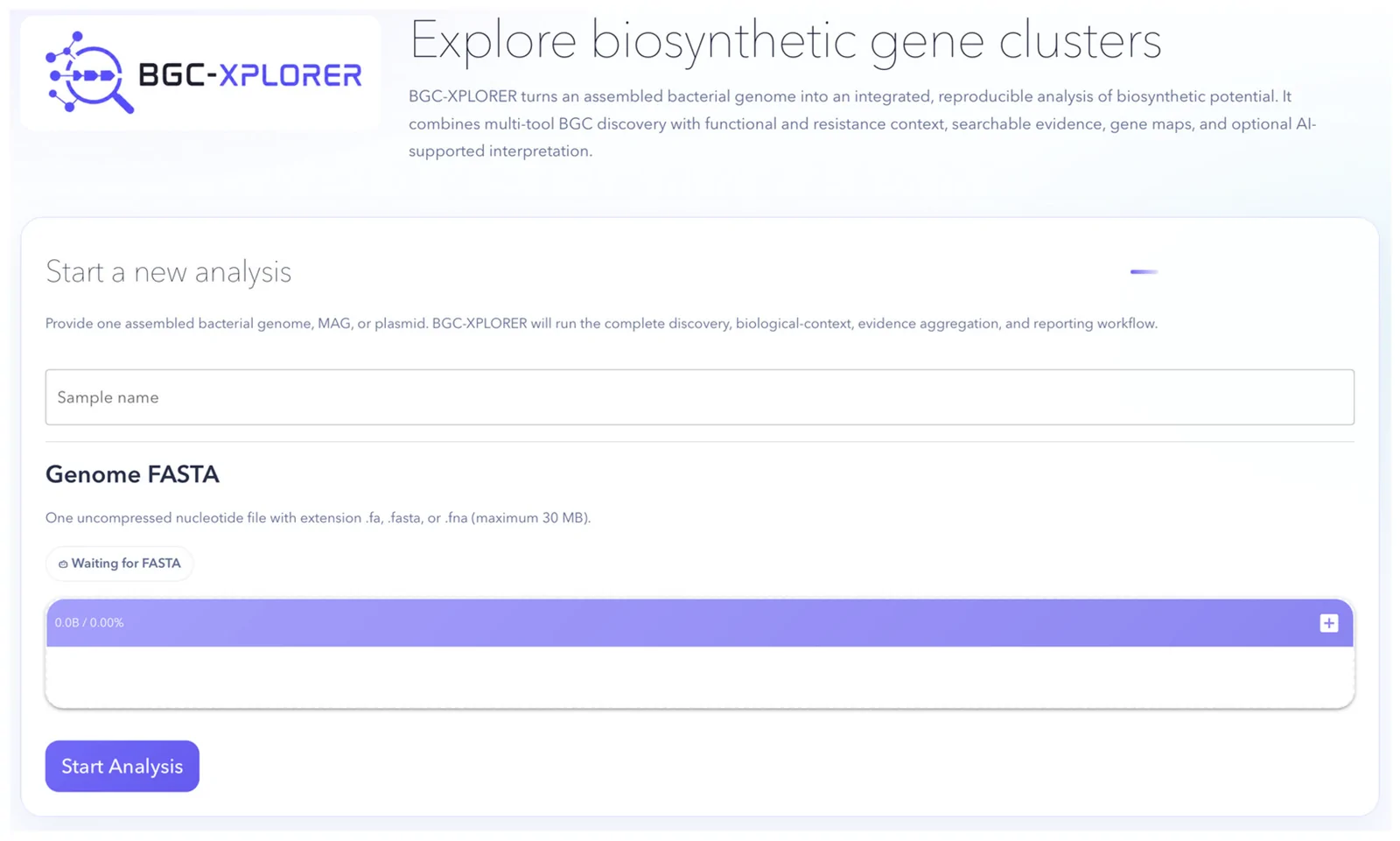
Analysis submission page of the BGC-XPLORER web interface.

**Figure 2:**
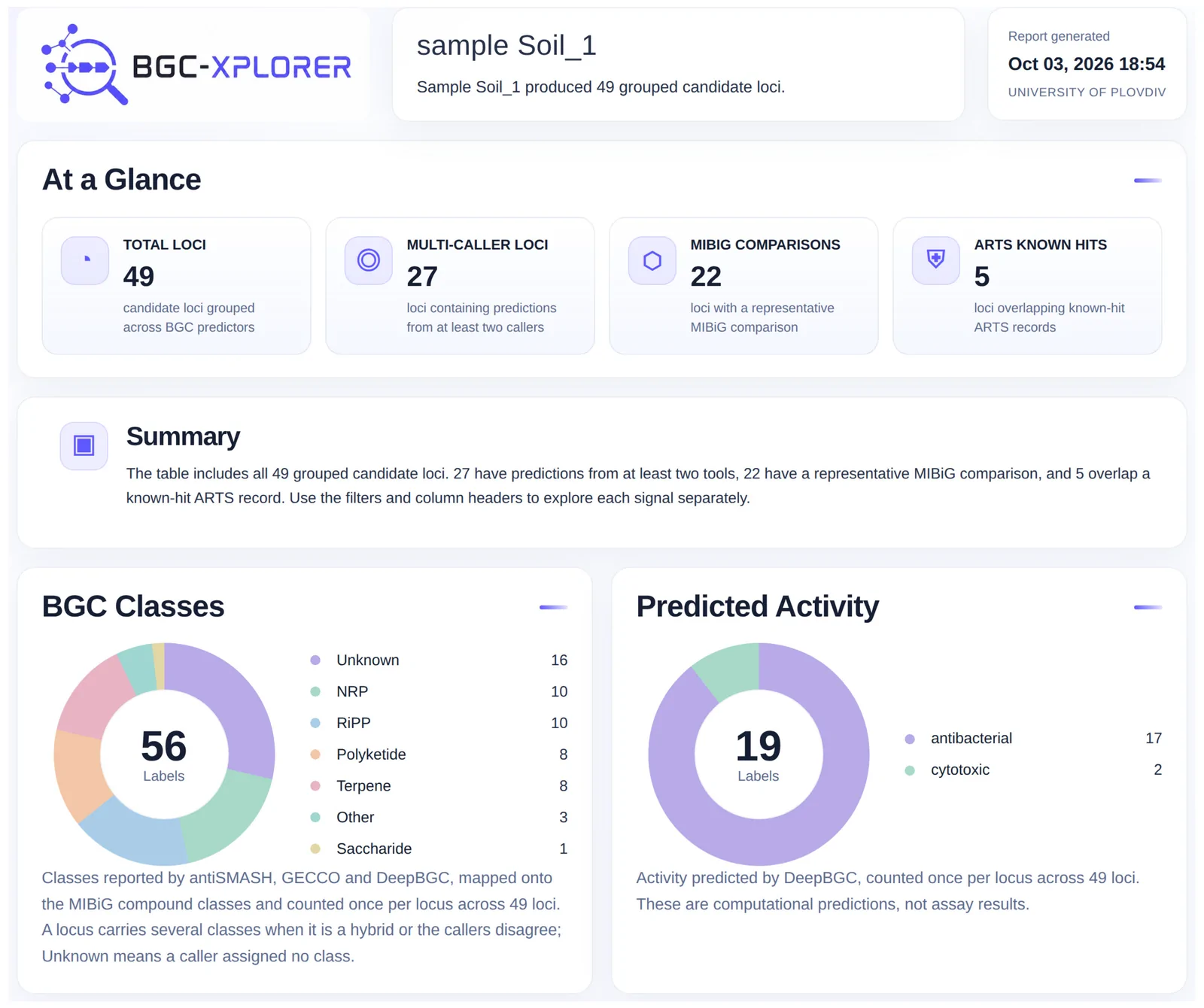
Report header, At a Glance metrics, and the MIBiG class and DeepBGC activity charts for *Streptomyces albidoflavus* STR-S001.

**Figure 3:**
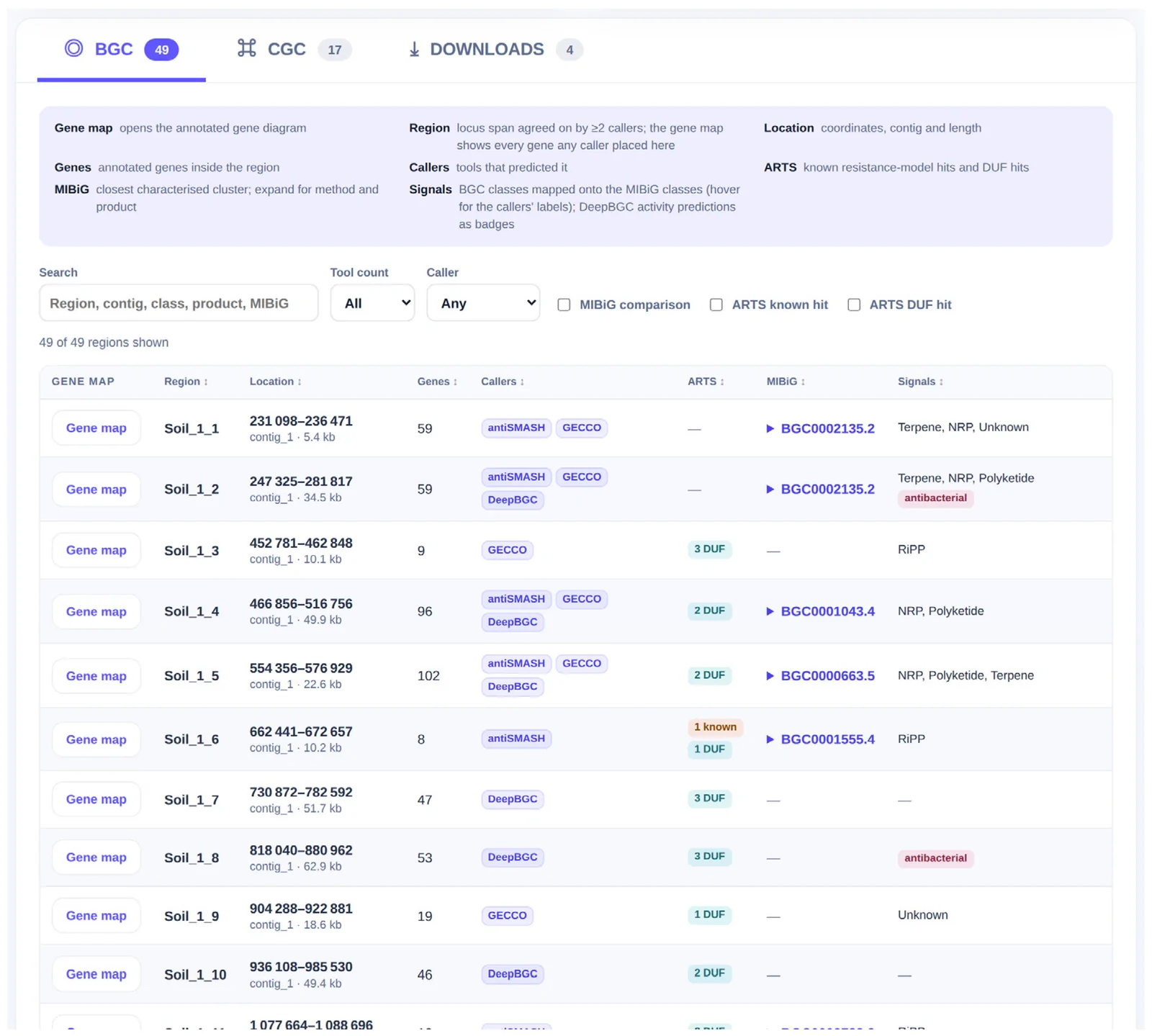
The searchable evidence table, showing the BGC tab with its column legend and filters.

**Figure 4:**
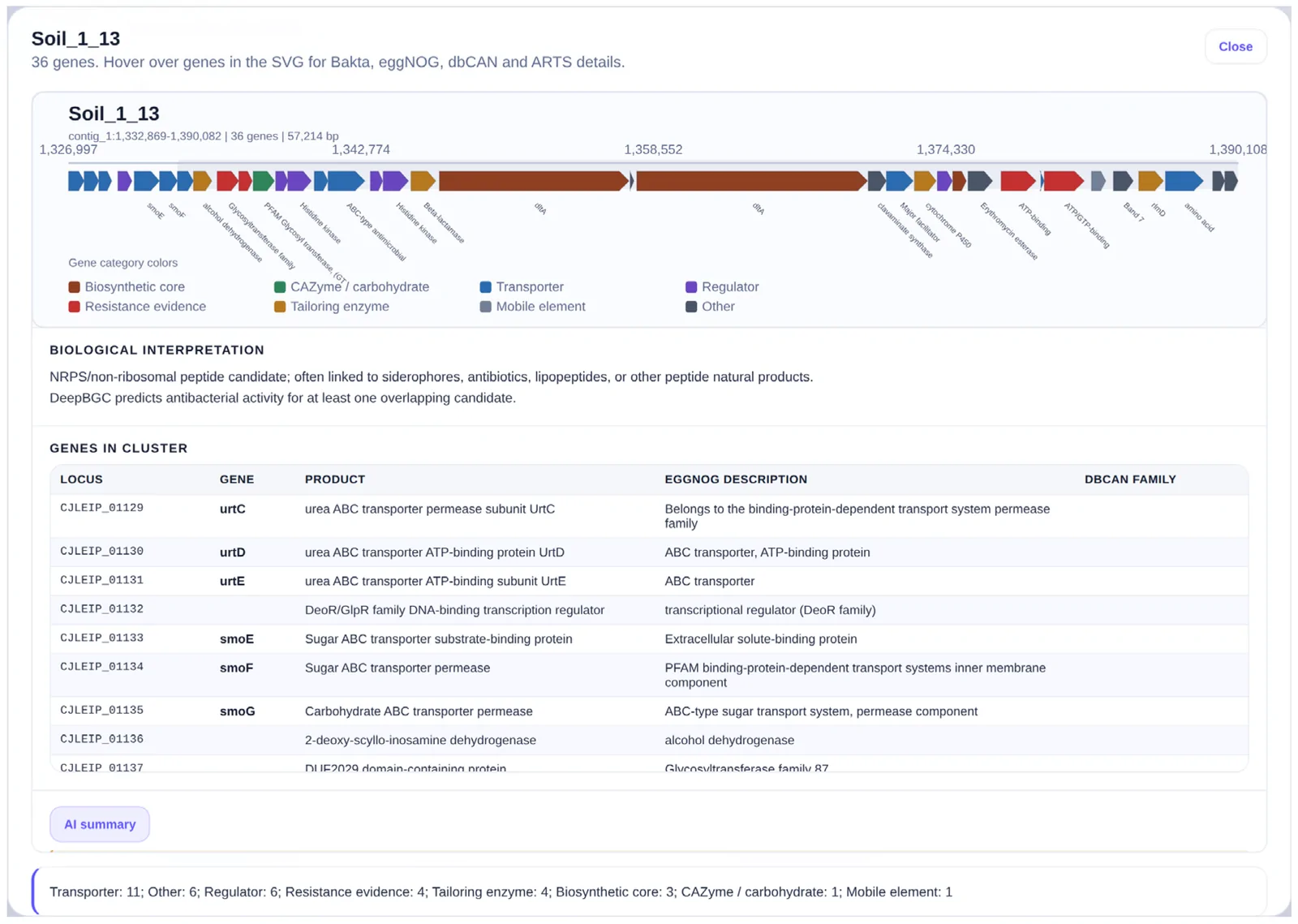
Gene map of locus Soil_1_13 (agreed span contig_1:1,332,869–1,390,082, drawn across contig_1:1,326,997–1,390,107) of strain STR-S001, with gene categories, biological interpretation and the per-gene Bakta, eggNOG and dbCAN table.

**Table 1:** Recovery and delimitation of the 42 MIBiG loci in four reference genomes, pooled.

Prediction Set	Detected (of 42)	Full/Partial/Missed	Fused	Median Jaccard	Median Gene Precision	Median Gene Recall	Median Gene F1
antiSMASH	40	36/4/2	4	0.458	0.362	1.000	0.515
GECCO	38	34/4/4	2	0.582	0.614	1.000	0.667
DeepBGC	32	28/4/10	4	0.163	0.213	1.000	0.220
BGC-XPLORER	41	37/4/1	2	0.524	0.478	1.000	0.626

Full: One prediction covers ≥95% of the MIBiG locus; partial: Predictions cover 10–95%; missed: <10%). Jaccard: Overlap divided by union of the MIBiG locus and the best-matching prediction, in bp. Gene precision: The fraction of the best-matching prediction’s genes that belong to the MIBiG locus; gene F1: Harmonic mean of gene precision and gene recall. Fused: The best-matching prediction also covers at least half of another reference locus on the same contig. Gene recall: The fraction of the MIBiG locus’s genes contained in the best-matching prediction; it is 1.000 in every prediction set, because a locus counts as recovered only when a prediction covers at least 95% of it, so gene precision is the informative half of the pair.

## Data Availability

The source code, documentation and container images of BGC-XPLORER are available at https://github.com/vebaev/BGC-XPLORER (accessed on 14 October 2026); the release described here is https://github.com/vebaev/BGC-XPLORER/releases/tag/v1.2.2 (accessed on 14 October 2026). The genome of *Streptomyces albidoflavus* STR-S001 is deposited in GenBank under accession CP158056.1 (https://www.ncbi.nlm.nih.gov/nuccore/CP158056.1, accessed on 14 October 2026). The provenance record of the run reported here (File S1) lists the tool versions and the checksums of the reference databases used.

## Supplementary Materials

The following supporting information can be downloaded at: https://www.mdpi.com/article/10.3390/cimb48101091/s1.
