# Supplementary material for: BGC-XPLORER: Exploring Biosynthetic Gene Clusters with Gene-Level Functional Annotation and AI-Assisted Summarization
Source: Curr Issues Mol Biol. 2026 Oct 21;48(10):1091. doi: 10.3390/cimb48101091 (PMC13702941; doi:10.3390/cimb48101091)
Supplement: Supplementary file 1 [file cimb-48-01091-s001.zip › File_S2_report_STR-S001.zip!/Soil_1_report/Soil_1.html]

BGC-XPLORER : sample Soil\_1


← Back to BGC-XPLORER

# sample Soil\_1

Sample Soil\_1 produced 49 grouped candidate loci.

Report generated

Oct 03, 2026 18:54

University of Plovdiv

## At a Glance

◔

Total loci

49

candidate loci grouped across BGC predictors

◎

Multi-caller loci

27

loci containing predictions from at least two callers

⬡

MIBiG comparisons

22

loci with a representative MIBiG comparison

⛨

ARTS known hits

5

loci overlapping known-hit ARTS records

▣

### Summary

The table includes all 49 grouped candidate loci. 27 have predictions from at least two tools, 22 have a representative MIBiG comparison, and 5 overlap a known-hit ARTS record. Use the filters and column headers to explore each signal separately.

## BGC Classes

**56**Labels

- Unknown16
- NRP10
- RiPP10
- Polyketide8
- Terpene8
- Other3
- Saccharide1

Classes reported by antiSMASH, GECCO and DeepBGC, mapped onto the MIBiG compound classes and counted once per locus across 49 loci. A locus carries several classes when it is a hybrid or the callers disagree; Unknown means a caller assigned no class.

## Predicted Activity

**19**Labels

- antibacterial17
- cytotoxic2

Activity predicted by DeepBGC, counted once per locus across 49 loci. These are computational predictions, not assay results.

## Cluster Gene Map

Close

### Biological interpretation

### Genes in cluster

| Locus | Gene | Product | eggNOG description | dbCAN family |
| --- | --- | --- | --- | --- |

AI summary

**Optional.** AI language models can make mistakes, and the text depends on the model chosen: check every statement against the evidence table. The loci, annotations and other results in this report do not depend on it.

◎BGC49⌘CGC17⤓DOWNLOADS4

Gene map
:   opens the annotated gene diagram

Region
:   locus span agreed on by ≥2 callers; the gene map shows every gene any caller placed here

Location
:   coordinates, contig and length

Genes
:   annotated genes inside the region

Callers
:   tools that predicted it

ARTS
:   known resistance-model hits and DUF hits

MIBiG
:   closest characterised cluster; expand for method and product

Signals
:   BGC classes mapped onto the MIBiG classes (hover for the callers' labels); DeepBGC activity predictions as badges

SearchTool countAll1 tool2 tools3 toolsCallerAnyantiSMASHGECCODeepBGCMIBiG comparisonARTS known hitARTS DUF hit

| Gene map | Region | Location | Genes | Callers | ARTS | MIBiG | Signals |
| --- | --- | --- | --- | --- | --- | --- | --- |
| Gene map | Soil\_1\_1 | 231 098–236 471contig\_1 · 5.4 kb | 59 | antiSMASHGECCO | — | BGC0002135.2 **Method**: ClusterCompare MIBiG | Terpene, NRP, Unknown |
| Gene map | Soil\_1\_2 | 247 325–281 817contig\_1 · 34.5 kb | 59 | antiSMASHGECCODeepBGC | — | BGC0002135.2 **Method**: ClusterCompare MIBiG | Terpene, NRP, Polyketideantibacterial |
| Gene map | Soil\_1\_3 | 452 781–462 848contig\_1 · 10.1 kb | 9 | GECCO | 3 DUF | — | RiPP |
| Gene map | Soil\_1\_4 | 466 856–516 756contig\_1 · 49.9 kb | 96 | antiSMASHGECCODeepBGC | 2 DUF | BGC0001043.4 **Method**: KnownClusterBlast  **Product**: SGR PTMs/SGR PTM Compound b/SGR PTM Compound c/SGR PTM Compound d | NRP, Polyketide |
| Gene map | Soil\_1\_5 | 554 356–576 929contig\_1 · 22.6 kb | 102 | antiSMASHGECCODeepBGC | 2 DUF | BGC0000663.5 **Method**: KnownClusterBlast  **Product**: hopene | NRP, Polyketide, Terpene |
| Gene map | Soil\_1\_6 | 662 441–672 657contig\_1 · 10.2 kb | 8 | antiSMASH | 1 known1 DUF | BGC0001555.4 **Method**: ClusterCompare MIBiG | RiPP |
| Gene map | Soil\_1\_7 | 730 872–782 592contig\_1 · 51.7 kb | 47 | DeepBGC | 3 DUF | — | — |
| Gene map | Soil\_1\_8 | 818 040–880 962contig\_1 · 62.9 kb | 53 | DeepBGC | 3 DUF | — | antibacterial |
| Gene map | Soil\_1\_9 | 904 288–922 881contig\_1 · 18.6 kb | 19 | GECCO | 1 DUF | — | Unknown |
| Gene map | Soil\_1\_10 | 936 108–985 530contig\_1 · 49.4 kb | 46 | DeepBGC | 2 DUF | — | — |
| Gene map | Soil\_1\_11 | 1 077 664–1 088 696contig\_1 · 11.0 kb | 12 | antiSMASH | 3 DUF | BGC0002788.2 **Method**: KnownClusterBlast  **Product**: 14-hydroxyisochainin | RiPP |
| Gene map | Soil\_1\_12 | 1 156 933–1 169 058contig\_1 · 12.1 kb | 13 | GECCO | 1 known1 DUF | — | Unknown |
| Gene map | Soil\_1\_13 | 1 332 869–1 390 082contig\_1 · 57.2 kb | 36 | antiSMASHGECCODeepBGC | 3 DUF | BGC0002358.3 **Method**: KnownClusterBlast  **Product**: cyclofaulknamycin | NRPantibacterial |
| Gene map | Soil\_1\_14 | 1 471 431–1 482 301contig\_1 · 10.9 kb | 26 | antiSMASHGECCO | — | BGC0002470.2 **Method**: KnownClusterBlast  **Product**: synechobactin C9/synechobactin C11/synechobactin 13/synechobactin 14/synechobactin 16/synechobactin A/synechobactin B/synechobactin C | Other, Unknown |
| Gene map | Soil\_1\_15 | 1 729 580–1 750 790contig\_1 · 21.2 kb | 41 | antiSMASHGECCODeepBGC | 2 DUF | BGC0001181.4 **Method**: KnownClusterBlast  **Product**: geosmin | Unknown, Terpeneantibacterial |
| Gene map | Soil\_1\_16 | 2 040 661–2 061 705contig\_1 · 21.0 kb | 25 | antiSMASH | 1 DUF | BGC0000262.5 **Method**: KnownClusterBlast  **Product**: prejadomycin/rabelomycin/gaudimycin C/gaudimycin D/UWM6/gaudimycin A | Terpene |
| Gene map | Soil\_1\_17 | 2 076 656–2 082 508contig\_1 · 5.9 kb | 20 | antiSMASHGECCO | — | BGC0000660.5 **Method**: KnownClusterBlast  **Product**: albaflavenone | Terpene |
| Gene map | Soil\_1\_18 | 2 399 833–2 432 469contig\_1 · 32.6 kb | 22 | DeepBGC | 2 DUF | — | Saccharide |
| Gene map | Soil\_1\_19 | 2 592 259–2 621 296contig\_1 · 29.0 kb | 55 | antiSMASHGECCODeepBGC | — | BGC0001145.5 **Method**: ClusterCompare MIBiG | RiPPantibacterial |
| Gene map | Soil\_1\_20 | 2 792 996–2 806 465contig\_1 · 13.5 kb | 13 | DeepBGC | 1 known | — | antibacterial |
| Gene map | Soil\_1\_21 | 2 824 714–2 836 467contig\_1 · 11.8 kb | 11 | GECCO | 1 DUF | — | Unknown |
| Gene map | Soil\_1\_22 | 2 861 769–2 872 845contig\_1 · 11.1 kb | 32 | GECCODeepBGC | 1 DUF | — | Unknown |
| Gene map | Soil\_1\_23 | 2 903 203–2 925 415contig\_1 · 22.2 kb | 24 | antiSMASHGECCO | — | BGC0000553.3 **Method**: CompariPPson  **Product**: Lanthipeptide: SRO15-2212 | RiPP |
| Gene map | Soil\_1\_24 | 2 949 769–2 998 269contig\_1 · 48.5 kb | 47 | antiSMASHGECCODeepBGC | 2 known1 DUF | BGC0000388.5 **Method**: KnownClusterBlast  **Product**: mannopeptimycin | NRPantibacterial |
| Gene map | Soil\_1\_25 | 3 043 783–3 055 798contig\_1 · 12.0 kb | 41 | GECCODeepBGC | 5 DUF | — | Unknown |
| Gene map | Soil\_1\_26 | 3 114 026–3 124 133contig\_1 · 10.1 kb | 9 | DeepBGC | 1 DUF | — | — |
| Gene map | Soil\_1\_27 | 3 158 437–3 173 204contig\_1 · 14.8 kb | 41 | GECCODeepBGC | 3 DUF | — | Unknown |
| Gene map | Soil\_1\_28 | 3 361 979–3 366 517contig\_1 · 4.5 kb | 32 | GECCODeepBGC | — | — | Unknown |
| Gene map | Soil\_1\_29 | 3 757 626–3 786 616contig\_1 · 29.0 kb | 32 | antiSMASHGECCODeepBGC | 5 DUF | BGC0001297.4 **Method**: KnownClusterBlast  **Product**: WS9326A/WS9326C/WS9326D | NRPantibacterial |
| Gene map | Soil\_1\_30 | 3 827 531–3 855 480contig\_1 · 27.9 kb | 25 | DeepBGC | 3 DUF | — | — |
| Gene map | Soil\_1\_31 | 3 898 355–3 930 212contig\_1 · 31.9 kb | 38 | DeepBGC | 1 known8 DUF | — | — |
| Gene map | Soil\_1\_32 | 4 060 268–4 163 383contig\_1 · 103.1 kb | 121 | antiSMASHGECCODeepBGC | 3 DUF | BGC0001792.5 **Method**: KnownClusterBlast  **Product**: surugamide A/surugamide D | NRP, RiPPantibacterialcytotoxic |
| Gene map | Soil\_1\_33 | 4 640 389–4 656 342contig\_1 · 16.0 kb | 27 | GECCODeepBGC | 1 DUF | — | NRP |
| Gene map | Soil\_1\_34 | 4 753 761–4 755 569contig\_1 · 1.8 kb | 3 | GECCO | — | — | Polyketide |
| Gene map | Soil\_1\_35 | 4 777 056–4 820 084contig\_1 · 43.0 kb | 37 | DeepBGC | 1 DUF | — | antibacterial |
| Gene map | Soil\_1\_36 | 4 901 213–4 915 178contig\_1 · 14.0 kb | 33 | antiSMASHGECCO | 2 DUF | BGC0000941.3 **Method**: KnownClusterBlast  **Product**: desferrioxamin B | Other, Unknown |
| Gene map | Soil\_1\_37 | 5 210 614–5 235 992contig\_1 · 25.4 kb | 34 | GECCODeepBGC | 1 DUF | — | Unknown |
| Gene map | Soil\_1\_38 | 5 288 071–5 299 166contig\_1 · 11.1 kb | 134 | GECCODeepBGC | — | — | Unknownantibacterial |
| Gene map | Soil\_1\_39 | 5 464 489–5 502 911contig\_1 · 38.4 kb | 30 | DeepBGC | — | — | antibacterial |
| Gene map | Soil\_1\_40 | 5 535 669–5 553 706contig\_1 · 18.0 kb | 16 | DeepBGC | 1 DUF | — | — |
| Gene map | Soil\_1\_41 | 5 599 507–5 616 849contig\_1 · 17.3 kb | 15 | DeepBGC | 1 DUF | — | cytotoxic |
| Gene map | Soil\_1\_42 | 5 813 848–5 825 367contig\_1 · 11.5 kb | 16 | antiSMASHGECCODeepBGC | — | BGC0000853.3 **Method**: KnownClusterBlast  **Product**: ectoine | Unknown, Otherantibacterial |
| Gene map | Soil\_1\_43 | 6 380 890–6 392 617contig\_1 · 11.7 kb | 13 | DeepBGC | 1 DUF | — | Polyketide |
| Gene map | Soil\_1\_44 | 6 525 343–6 534 041contig\_1 · 8.7 kb | 7 | GECCO | — | — | RiPP |
| Gene map | Soil\_1\_45 | 6 569 492–6 598 955contig\_1 · 29.5 kb | 50 | antiSMASHGECCODeepBGC | 1 DUF | BGC0001456.3 **Method**: KnownClusterBlast  **Product**: isorenieratene | Terpene, RiPP |
| Gene map | Soil\_1\_46 | 6 679 935–6 691 963contig\_1 · 12.0 kb | 26 | antiSMASHDeepBGC | — | BGC0002115.3 **Method**: KnownClusterBlast  **Product**: streptamidine | RiPPantibacterial |
| Gene map | Soil\_1\_47 | 6 733 580–6 758 544contig\_1 · 25.0 kb | 171 | antiSMASHGECCODeepBGC | 1 DUF | BGC0001310.5 **Method**: KnownClusterBlast  **Product**: naringenin | Polyketide, Unknownantibacterial |
| Gene map | Soil\_1\_48 | 6 762 952–7 035 108contig\_1 · 272.2 kb | 154 | antiSMASHGECCODeepBGC | 3 DUF | BGC0000034.5 **Method**: KnownClusterBlast  **Product**: candicidin | Polyketide, Terpene, NRP, RiPPantibacterial |
| Gene map | Soil\_1\_49 | 7 041 821–7 049 010contig\_1 · 7.2 kb | 154 | GECCODeepBGC | — | — | Polyketide, Unknownantibacterial |

Gene map
:   opens the annotated gene diagram

CGC
:   dbCAN carbohydrate gene cluster id

Location
:   coordinates, contig and length

Genes
:   annotated genes inside the cluster

CAZymes
:   carbohydrate-active enzyme genes

Signatures
:   signature genes: CAZyme, transporter, transcription factor, sulfatase or peptidase

Predicted substrate
:   the carbohydrate the cluster likely acts on

Support source
:   dbCAN-PUL homology, dbCAN-sub majority vote, or both

dbCAN-sub substrate
:   the dbCAN-sub call on its own

| Gene map | CGC | Location | Genes | CAZymes | Signatures | Predicted substrate | Support source | dbCAN-sub substrate |
| --- | --- | --- | --- | --- | --- | --- | --- | --- |
| Gene map | CGC1 | 62 974–70 239contig\_1 · 7.3 kb | 5 | 2 | 5 | xylan | dbCAN-PUL only |  |
| Gene map | CGC6 | 416 021–425 009contig\_1 · 9.0 kb | 7 | 1 | 6 | xylan | dbCAN-PUL only |  |
| Gene map | CGC8 | 711 046–721 034contig\_1 · 10.0 kb | 6 | 2 | 5 | levoglucosan | dbCAN-PUL only |  |
| Gene map | CGC9 | 852 429–867 171contig\_1 · 14.7 kb | 9 | 2 | 8 | xylan | dbCAN-PUL only |  |
| Gene map | CGC11 | 932 151–935 000contig\_1 · 2.9 kb | 2 | 1 | 2 | starch | dbCAN-PUL only |  |
| Gene map | CGC20 | 1 428 863–1 432 344contig\_1 · 3.5 kb | 3 | 1 | 3 | starch | dbCAN-PUL only |  |
| Gene map | CGC24 | 1 855 133–1 872 652contig\_1 · 17.5 kb | 9 | 4 | 6 | glycogen \| alpha-glucan | dbCAN-PUL + dbCAN-sub | alpha-glucan |
| Gene map | CGC25 | 1 932 533–1 941 778contig\_1 · 9.2 kb | 7 | 2 | 6 | cellulose,chitin | dbCAN-sub only | cellulose,chitin |
| Gene map | CGC28 | 2 064 215–2 075 799contig\_1 · 11.6 kb | 9 | 1 | 7 | chitin | dbCAN-PUL only |  |
| Gene map | CGC34 | 2 537 576–2 544 010contig\_1 · 6.4 kb | 5 | 1 | 5 | beta-glucan | dbCAN-PUL only |  |
| Gene map | CGC46 | 4 591 458–4 597 324contig\_1 · 5.9 kb | 5 | 2 | 5 | beta-glucan | dbCAN-PUL only |  |
| Gene map | CGC52 | 4 885 499–4 892 263contig\_1 · 6.8 kb | 5 | 1 | 5 | beta-glucan | dbCAN-PUL only |  |
| Gene map | CGC54 | 5 031 906–5 044 764contig\_1 · 12.9 kb | 8 | 4 | 6 | hostglycan | dbCAN-sub only | hostglycan |
| Gene map | CGC60 | 5 453 674–5 464 361contig\_1 · 10.7 kb | 7 | 3 | 7 | starch \| alpha-glucan | dbCAN-PUL + dbCAN-sub | alpha-glucan |
| Gene map | CGC71 | 6 504 990–6 508 426contig\_1 · 3.4 kb | 3 | 1 | 3 | beta-glucan | dbCAN-PUL only |  |
| Gene map | CGC72 | 6 528 998–6 540 515contig\_1 · 11.5 kb | 8 | 3 | 5 | xylan | dbCAN-sub only | xylan |
| Gene map | CGC73 | 6 639 533–6 652 791contig\_1 · 13.3 kb | 11 | 2 | 10 | xylan | dbCAN-PUL only |  |

- ⤓BGC tableOne row per grouped candidate locus, as shown in the BGC tabregion\_evidence.tsv · 48.4 KB
- ⤓CGC tableAll dbCAN carbohydrate gene clusters, including those without a resolved substratedbcan.cgc.tsv · 11.4 KB
- ⤓Bakta annotationEvery annotated feature: locus tags, coordinates, productsSoil\_1.features.tsv · 1.3 MB
- ⤓eggNOG annotationOrthology assignments with EC, KEGG KO and COG categorieseggnog.annotations.tsv · 2.1 MB

## Reproducibility

Version: **v1.2.2** · Commit: `0d44d085aeb5a75512c052d3ca24c68bc6da2568` · Bakta database: **full 6.0** · ARTS reference: **actinobacteria** · Active AI model: `Checking active model...`

BGC-XPLORER ReportSample: Soil\_1Oct 03, 2026 18:54
